# Comparison of Rapid Descriptive Sensory Methods Applied to Consumers in the Evaluation of Muffins

**DOI:** 10.3390/foods14162898

**Published:** 2025-08-21

**Authors:** Reynaldo J. Silva-Paz, Humberto A. Avilés Pérez, Thalia A. Rivera-Ashqui, Carmen R. Apaza-Humerez

**Affiliations:** 1 Escuela de Ingeniería en Industrias Alimentarias, Departamento de Ingeniería, Universidad Nacional de Barranca, Av. Toribio de Luzuriaga N° 376 Mz J. Urb. La Florida, Barranca 15169, Peru; 2 Escuela Profesional de Ingeniería de Industrias Alimentarias, Facultad de Ingeniería y Arquitectura, Universidad Peruana Unión, Km 19 Carretera Central, Ñaña, Lima 15457, Peru; alex_av@upeu.edu.pe (H.A.A.P.); thaliarivera@upeu.edu.pe (T.A.R.-A.)

**Keywords:** perception, descriptive, sensory, CATA, flash profile, pivot

## Abstract

Sensory evaluation is essential to understand consumer perception. This study compared three descriptive methods (Check-All-That-Apply (CATA), Flash Profile, and Pivot Profile) to characterize muffins formulated with alternative flours (purple corn and amaranth) in comparison to a wheat-based control. Six formulations (T0–T5) were evaluated: CATA and Pivot Profile were applied with 100 consumers, while Flash Profile was conducted with 15 panelists. Multivariate statistical analyses were used: correspondence analysis for CATA and Pivot, and Generalized Procrustes Analysis for Flash Profile. All three methods showed high discriminative power: CATA explained 94.36% of the variance, identifying three main groups; Flash Profile explained 63.88%, highlighting differences in texture and aroma; and Pivot Profile explained 81.10%, revealing complex interactions among sensory attributes. Sample T1 (100% purple corn) showed a distinctive sensory profile (bitter and dry), while samples T2 to T5 presented intermediate characteristics. The RV coefficient confirmed significant congruence between the methods. CATA effectively identified relevant sensory differences, Pivot Profile generated descriptors in relation to a control sample, and Flash Profile enabled exploratory analysis. The choice of method depends on the study objective, with each approach offering complementary sensory information.

## 1. Introduction

Sensory evaluation is a fundamental tool in the food industry, as it enables the characterization of key attributes that determine consumer acceptance and preference [1]. For decades, Quantitative Descriptive Analysis (QDA) has been regarded as the gold standard due to its methodological rigor and capacity to generate highly reproducible data [2]. However, this approach presents significant limitations in industrial contexts, where rapid and cost-effective assessments are prioritized. The training of expert panels demands considerable investments of time (between 60 and 100 h) and financial resources [3], which has driven the development of Rapid Descriptive Methods (RDMs) as a viable alternative.

In recent years, techniques such as Check-All-That-Apply (CATA), Flash Profile, and Pivot Profile have revolutionized sensory evaluation by delivering reliable results with untrained panels and within significantly shorter time frames [4]. These methods have shown particular usefulness across various food matrices, ranging from dairy products to snacks and beverages [5,6]. However, their application in baked goods—particularly muffins—has been scarcely documented in the scientific literature, despite the growing relevance of these products in the global food market [7].

Muffins represent a particularly interesting case for sensory evaluation due to their structural complexity and the critical importance of attributes such as fluffiness, moisture, and the sweet–acidic balance in consumer experience [8]. The industry faces the challenge of efficiently characterizing these attributes, especially during new product development and quality control processes. While the CATA method has shown excellent performance in evaluating products with less complex sensory profiles [9], Flash Profile has proven superior in matrices where interactions between texture and flavor are critical [6]. Pivot Profile, conversely, has emerged as a valuable tool for discriminating between products with subtle differences, as is often the case with variations within the same product line [10], which can be complemented with discriminative techniques [11].

The optimal choice of sensory method depends on multiple factors, including the type of product, the critical attributes to be evaluated, and the available resources. Recent studies have emphasized that while CATA offers advantages in simplicity and speed [12], Flash Profile provides more detailed insights into relationships among descriptors [13]. Pivot Profile, meanwhile, has shown particular sensitivity in detecting minimal differences between samples [10]. Nonetheless, there is currently no consensus on which method offers the best balance between efficiency and precision when applied to baked products with complex structures, such as muffins.

This study addresses a relevant gap in applied sensory science by comparing rapid descriptive methods for the evaluation of muffins. The findings have direct implications for the baked goods industry, which accounts for more than 15% of the global processed food market [14], by supporting informed decisions in product development and quality control processes. In a context where speed and efficiency are critical to competitiveness, this study provides evidence to guide the selection of appropriate sensory methods without compromising data quality. Furthermore, the results offer valuable tools for optimizing resources in the food industry and lay the groundwork for future research on the application of rapid techniques to other complex food matrices, thus contributing to the advancement of sensory evaluation knowledge. In this context, the objective of this work is to compare the CATA, Flash Profile, and Pivot Profile methods for the sensory characterization of muffins.

## 2. Materials and Methods

### 2.1. Muffin Preparation

**Figure 1:**
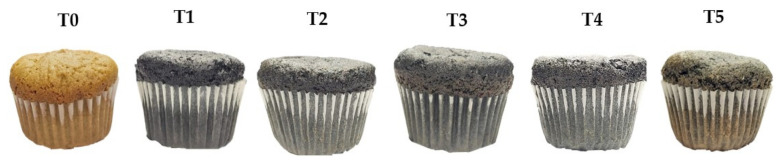
Samples used for sensory evaluation development. T0: control sample (100% wheat flour); T1: 100% purple corn flour (PCF); T2: 25% PCF/75% KF; T3: 50% PCF/50% KF; T4: 75% PCF/25% KF; and T5: 100% kiwicha flour (KF).

**Figure 2:**
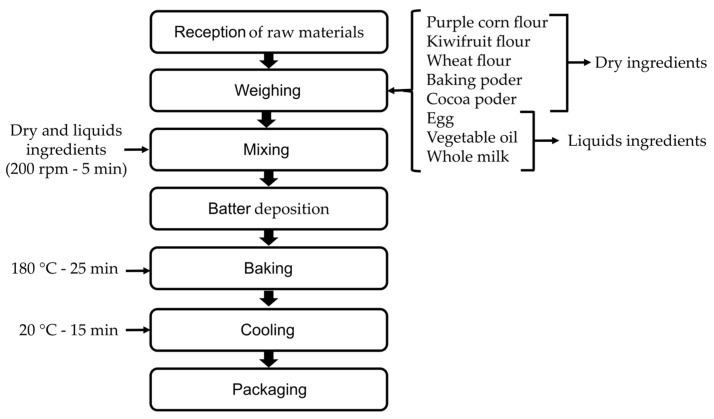
Flow diagram of the muffin production process.

Six muffin variants were formulated by partially or totally replacing wheat flour with alternative flours (purple corn and kiwicha), following standardized baking protocols [7]. The formulations included the following: one control sample with 100% wheat flour (T0); one sample with 100% purple corn flour (T1); one sample with 100% kiwicha flour (T5); and three mixed formulations with varying proportions of both alternative flours—25/75%, 50/50%, and 75/25% purple corn/kiwicha flour for T2, T3, and T4, respectively (Figure 1). The base formulation, common to all variants, consisted of flour (25.5%), white sugar (16.5%), whole milk (22%), eggs (16.5%), baking powder (1.8%), cocoa powder (1.2%), and vegetable oil (16.5%), as established in previous optimization studies of baked products [8]. Preparation involved homogeneous mixing of dry ingredients, followed by gradual incorporation of liquids, mixing at 250 rpm for 5 min, and baking at 180 °C for 25 min using standardized molds to ensure identical processing conditions across all samples (Figure 2).

### 2.2. Sensory Tests

#### 2.2.1. Consumers

A total of 100, 80, and 15 consumers participated in the CATA, Pivot Profile, and Flash Profile methods, respectively. Participants were recruited from a university population and met the inclusion criteria: regular consumption of baked goods (≥once per week) and absence of relevant food allergies [15]. Evaluation sessions were conducted in individual booths under standardized lighting conditions (D65), following international guidelines for sensory testing [16].

Each participant received 20 ± 1 g portions of each muffin formulation, served on disposable plates coded with random three-digit numbers and presented using a balanced design to control order and fatigue effects [17]. A 60 s interval was implemented between samples, during which participants rinsed with still mineral water at 25 °C to neutralize the palate [18]. All participants provided informed consent and received standardized verbal instructions to ensure understanding of the procedure. The protocol was approved by the Institutional Ethics Committee (Reg. No. 2025-CEFIAINDALI-002, 21 February 2025).

#### 2.2.2. Check-All-That-Apply (CATA) Analysis

The CATA test followed validated methodologies from recent sensory evaluation studies. Prior to the main study, a pilot test with 30 consumers was conducted to select the most relevant descriptors through free listing and semantic grouping. The final test included 20 descriptors grouped into four main categories: color attributes (dark); texture attributes (spongy, lumpy, gummy, dry, soft, moist, sticky, gritty, porous, or greasy); aroma attributes (corn aroma, vanilla aroma, or chocolate aroma); and flavor attributes (sweet, low sweetness, bland, bitter, mild flavor, chocolate flavor, or savory) [19,20]. This categorization allowed for a structured and efficient sensory assessment. Each participant evaluated the six samples—coded with three-digit random numbers and presented in balanced monadic random order—by checking all applicable descriptors on the evaluation form.

#### 2.2.3. Flash Profile

The Flash Profile method was carried out in three consecutive phases adapted from standardized protocols. During the initial 20 min phase, participants received training in descriptive techniques and the use of physical references. In the second phase, a guided group session, panelists generated a list of descriptors through direct comparison of all samples. The third and final phase consisted of an independent session in which participants ranked the samples by intensity for each descriptor, scoring them simultaneously using the terms generated during the previous session [13]. All consumers received all samples at once (global presentation), coded with random three-digit numbers.

#### 2.2.4. Pivot Profile

The Pivot Profile evaluation used sample T0 (100% wheat flour) as the reference. The protocol included a familiarization phase in which participants compared the pivot sample with itself to establish a sensory baseline. In the main sessions, each panelist evaluated the five experimental samples against the pivot, following a complete balanced design. Sensory differences were recorded using response forms to identify attributes perceived as more or less intense than the reference [21]. All samples were presented in random order under controlled lighting and identified with three-digit random codes, with strict palate-cleansing protocols between evaluations.

### 2.3. Comparison of Sensory Methods Using the RV Coefficient

To compare the congruence among the results obtained with the different sensory methods (CATA, Flash Profile, and Pivot Profile), the RV coefficient was calculated using the method of Escoufier [22]. This multivariate correlation measure evaluates the similarity between spatial configurations derived from different analytical techniques. The analysis was performed on the data matrices generated by each method after standardization and transformation to ensure comparability. RV coefficient values range from 0 (no correlation) to 1 (perfect correlation), allowing for the quantification of agreement among the multidimensional sample representations obtained by each technique. This methodological approach, validated in recent studies comparing sensory methods [23], provides a robust measure of agreement and helps identify potential discrepancies in sensory characterization.

### 2.4. Statistical Analysis

Specific statistical techniques were applied to the data obtained from each sensory method. CATA data were analyzed using frequency analysis of descriptor selection and Multiple Correspondence Analysis (MCA) to visualize relationships between samples and attributes [20]. For the Flash Profile, the ranking data were processed using Generalized Procrustes Analysis (GPA) to obtain consensus coordinates of the samples [24]. Pivot Profile results were analyzed using textual content analysis to group semantically similar descriptors, followed by the construction of a sample × attribute contingency matrix and correspondence analysis to generate comparative sensory profiles.

Method comparison was conducted by calculating the RV coefficient between the distance matrices obtained from each technique, complemented by a permutation test (*n* = 1000) to assess statistical significance [25]. All analyses were performed using XLSTAT 2023 for general statistics. RV coefficient analysis was conducted in R (v4.2.1) using the FactoMineR package version 2.10.

## 3. Results

### 3.1. Consumers

Table 1 presents the sociodemographic distribution of participants in each sensory evaluation method. Slight differences in gender composition were observed, with a female predominance in CATA (53%) and Pivot Profile (53.33%), while the Flash Profile method recorded higher male participation (53.75%). These findings align with existing scientific evidence reporting gender-based differences in sensory perception, particularly in the detection and perceived intensity of attributes such as sweetness (10–20% lower detection threshold in women) and texture (greater female sensitivity to variations in viscosity and granularity) [26,27]. The balanced gender distribution observed (range 47–53.8%) meets international recommendations for consumer sensory testing, which suggest gender-balanced panels to minimize bias in product characterization [15].

The age group analysis revealed minimal differences among young adults (18–30 years), particularly in CATA (84%) and Pivot Profile (80%). In contrast, the Flash Profile method had a higher representation of the 31–39 age group (26.25%), possibly due to the increased cognitive demand of this methodology. These results are relevant given the documented physiological changes in sensory perception: a 30–40% reduction in taste buds between the ages of 20 and 60, and a progressive decline in olfactory sensitivity [28]. The limited participation of older adults (>40 years, <8% across all methods) restricts the generalizability of results to this demographic, known for distinctly different preference patterns, especially in baked goods [29]. This limitation should be considered when interpreting the data, as longitudinal studies have shown up to 1.5-point variations on 9-point hedonic scales between extreme age groups for similar products [30].

### 3.2. Sensory Evaluation

#### 3.2.1. Check-All-That-Apply (CATA) Method

The results obtained using the CATA methodology provide valuable information on the sensory perception of muffins made with alternative flours. The high explained variance (94.36%) confirms the suitability of the method for characterizing this type of product, in line with what was reported by Ares et al. [9] in studies on baked goods. It is particularly noteworthy that CATA was able to discriminate between samples primarily based on textural attributes, such as lumpy and dry-grainy, rather than basic taste characteristics. This suggests that flour substitutions predominantly modify the structure of the food matrix.

These findings are consistent with previous research highlighting CATA’s sensitivity in detecting differences in products with complex formulations [31]. However, the low discrimination observed for the sweet attribute may indicate a limitation of the method in capturing subtle variations in sweetness or that the formulations evaluated did not differ significantly in this parameter. This contrasts with findings by Gómez et al. [7], who reported changes in sweetness perception in products with partial flour substitution, suggesting that the impact depends on the specific type of alternative flour used.

**Figure 3:**
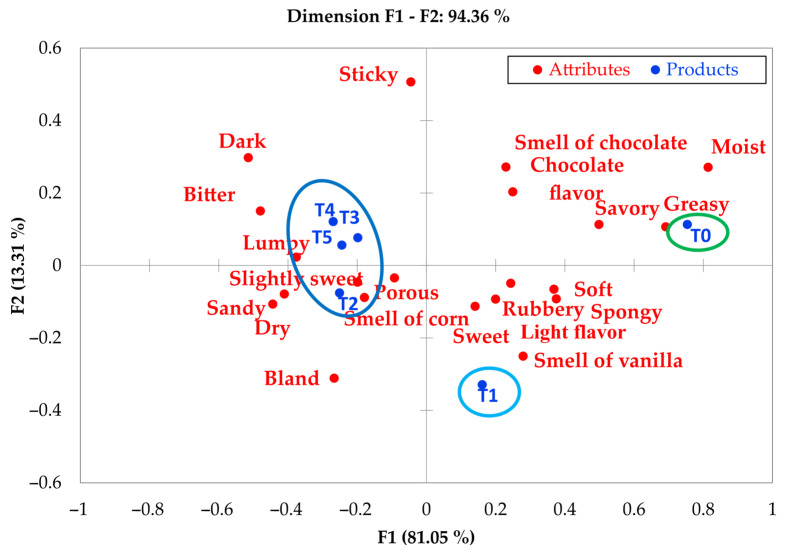
Sensory map of the attribute samples obtained by the CATA method. T0: control sample, 100% wheat flour; T1: 100% purple corn flour (PCF); T2: 25% PCF/75% KF; T3: 50% PCF/50% KF; T4: 75% PCF/25% KF; and T5: 100% kiwicha flour (KF).

Figure 3 presents the sensory map generated using the CATA method, where three main clusters of samples were identified. Samples T2, T3, T4, and T5 were significantly associated with descriptors such as lumpy, dry-sandy, and smell of purple corn, reflecting the typical characteristics of gluten-free flour blends where the product structure is altered [7]. In contrast, the control sample (T0) was associated with attributes like greasy, moist, and savory, which are typical of products made with conventional wheat flour. Sample T1 (100% purple corn flour) exhibited a unique sensory profile, characterized by attributes such as light flavor, sweet, and smell of vanilla, likely related to its antioxidant compounds [8]. The descriptors with the highest discriminative power were greasy (cos^2^ = 0.72) and lumpy (cos^2^ = 0.68), while sweet showed no significant discriminative power (cos^2^ < 0.15), reinforcing the idea that the differences between formulations were focused more on textural and aromatic attributes rather than sweetness ones. The clustering of T2, T3, T4, and T5 suggests that the CATA method is capable of identifying intermediate sensory profiles in mixed-flour formulations, although it presents limitations in discriminating between similar proportions (e.g., 25–75% vs. 50–50%). This limitation could be overcome by incorporating intensity scales, as proposed in the RATA (Rate-All-That-Apply) variant [2,5]. The CATA method allows the identification of sensory deviations from the control product (T0), especially in critical attributes for likeability, such as texture and moisture.

#### 3.2.2. Flash Profile Method

**Figure 4:**
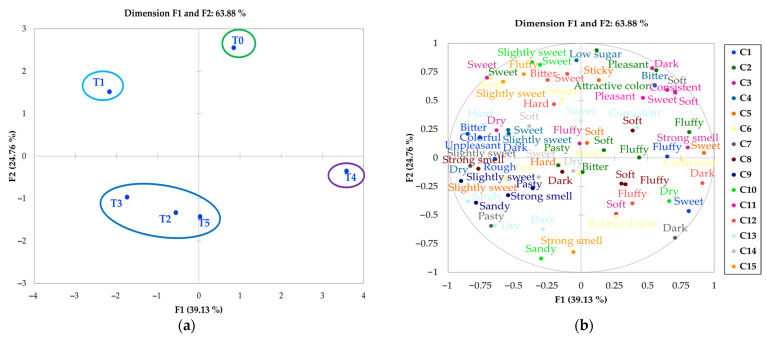
Sensory map of the samples (**a**) and attributes (**b**) using the Flash Profile method with a consumer panel. T0: control sample, 100% wheat flour; T1: 100% purple corn flour (PCF); T2: 25% PCF/75% KF; T3: 50% PCF/50% KF; T4: 75% PCF/25% KF; and T5: 100% kiwicha flour (KF). C1 to C15 correspond to individual consumer participants.

Figure 4 presents the results obtained using the Flash Profile method. The sensory map of the samples (Figure 4a) and the configuration of the attributes (Figure 4b) were generated through Generalized Procrustes Analysis (GPA). The first two components explained 63.88% of the total variance, a value lower than that obtained with CATA, but within the range reported for Flash Profile applications in complex matrices (60–70%) [30,32]. The spatial configuration revealed four well-defined clusters. Sample T1 (100% purple corn) was distinctly positioned in the upper-left quadrant, associated with descriptors such as sweet, soft, dry, and toasted notes, which aligns with the unique phenolic profile of this flour [7]. Samples T2, T3, and T5 formed a compact group in the lower-left quadrant, linked to attributes such as intense aroma, balanced flavor, sandy and pasty textures, and reduced sweetness, suggesting that intermediate proportions of alternative flours (25–50%) produce similar sensory profiles. In contrast, T4 (75% purple corn) showed a more differentiated profile, sharing some attributes with the previous group but characterized by descriptors like dark, dense, sweet, and consistent. The control sample (T0) was located in the upper-right quadrant, associated with typical attributes of conventional muffins, such as spongy, sticky, and pleasant.

From a methodological perspective, these results highlight both the strengths and limitations of the Flash Profile method. On one hand, it demonstrated greater sensitivity than CATA in detecting differences in complex attributes such as aroma and specific textural characteristics, especially between extreme formulations (T0 vs. T1). On the other hand, its lower capacity to describe differences among formulations with similar proportions (T2, T3, and T5) suggests that it could benefit from incorporating structured intensity scales in future studies [12].

These findings have relevant implications for product development. The clear differentiation of the control sample (T0) reflects the technological challenges associated with total wheat flour substitution, while the clustering of intermediate formulations suggests that small variations in the proportion of alternative flours (25–50%) may not result in significant sensory differences, potentially simplifying reformulation processes. The distinct positioning of T1 further reinforces the need for specific adjustments in formulations with 100% purple corn, particularly regarding texture and aroma attributes.

In total, the consumer panel generated 23 descriptors distributed across the categories of flavor (8), aroma (1), color (3), and texture (11), including the following: tasty, sweet, bitter, low in sugar, balanced flavor, not sweet, pleasant, unpleasant, intense aroma, colorful, dark, attractive color, consistent, soft, spongy, hard, sticky, tender, dry, pasty, sandy, crumbly, and rough. The Flash Profile method allows for simultaneous evaluation and rapid representation of muffins in sensory space, in accordance with what has been reported in other studies on similar products [33,34].

#### 3.2.3. Pivot Profile Method

**Figure 5:**
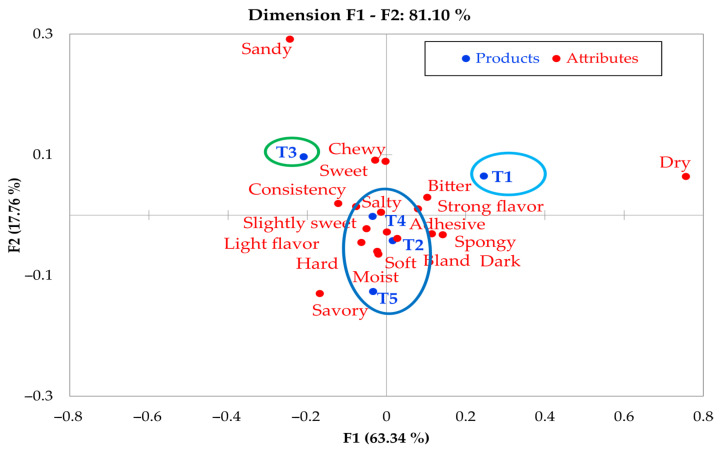
Sensory map of the samples using the pivot profile method. T1: 100% purple corn flour (PCF); T2: 25% PCF/75% KF; T3: 50% PCF/50% KF; T4: 75% PCF/25% KF; and T5: 100% kiwicha flour (KF).

The analysis using the Pivot Profile method proved to be an effective tool for characterizing the complex sensory interactions present in the muffin formulations, explaining 81.10% of the total variance through its two main components (F1 = 63.34% and F2 = 17.76%) (Figure 5). These results are similar to those reported by Thuillier et al. [10], highlighting the sensitivity of the method to detect subtle differences between samples.

The marked separation of sample T1 in the sensory space—associated with descriptors such as dry and bitter—corroborates the findings obtained with the CATA and Flash Profile methods, although it had also been described as dry, sweet, light flavor, and smell of vanilla, reaffirming the distinctive sensory character of the 100% purple corn flour formulation. In turn, sample T3 was positioned as sweet and chewy, suggesting a balanced profile, likely attributable to the specific flour combination used in its formulation. The grouping of samples T2, T4, and T5 was particularly interesting, as the Pivot Profile method revealed the coexistence of seemingly contradictory attributes (e.g., hard vs. moist and savory vs. bland-dark). This phenomenon, described in complex food systems [35], is attributed to the structural heterogeneity of gluten-free muffins, in which moister regions coexist with dense crumb areas; the differential release of volatile compounds during mastication; and possible interactions between purple corn phenolics and amaranth proteins.

These results reflect the fundamental differences between the methods: Flash Profile generates descriptors based on the spontaneous vocabulary of the consumer; CATA relies on a closed list of predefined attributes, which may limit vocabulary richness; and Pivot Profile uses a reference sample or pivot, allowing assessors to describe relative attributes without categorical constraints, thus capturing greater sensory complexity.

These observations underline one of the main advantages of the Pivot Profile method over more structured approaches: its ability to capture sensory multidimensionality without imposing predefined categories [10]. From a methodological perspective, the results support the recommendations given by Stone et al. [36] regarding the importance of applying complementary analytical approaches. While CATA and Flash Profile provided a general overview of differences among samples, Pivot Profile allowed the identification of subtler nuances, particularly regarding attribute interactions.

However, the relatively low contribution of the second component (F2 = 17.76%) suggests that some secondary sensory dimensions may be underrepresented. This issue could be addressed in future studies through the incorporation of temporal evaluation methods, such as TCATA (Temporal Check-All-That-Apply) or TDS (Temporal Dominance of Sensations), which allow for a more dynamic analysis of sensory perception.

### 3.3. Comparison of Rapid Descriptive Methods

Table 2 presents a comparison of the three descriptive methods (CATA, Flash Profile, and Pivot Profile) using the RV coefficient, which measures the similarity of results across the different methods for the same set of samples (T0 to T5). The RV coefficient is used to assess concordance between sensory spaces generated by different analytical techniques.

The RV coefficient between CATA and Flash Profile (0.525) suggests moderate agreement in the way the two methods describe sample positioning, indicating a reasonable degree of similarity in the sensory profiles obtained. However, significant differences were noted in how each method positions samples, as shown by the values of the first two components (F1 and F2). For instance, sample T0 showed greater alignment between CATA and Flash Profile (RV coefficient of 0.754 for F1 in CATA), but discrepancies were more pronounced for samples such as T2 and T5.

The RV coefficient between Flash Profile and Pivot Profile (0.249) was lower, indicating weak correspondence. This suggests that the sensory representations generated by these two methods are notably different, possibly reflecting differences in how each technique maps the samples in multidimensional sensory space. In contrast, the RV coefficient between CATA and Pivot Profile (0.741) indicates strong concordance, showing that despite methodological differences, the sensory positioning of samples—particularly T0 and T1—was relatively consistent.

These results underscore that while the three methods share certain points of agreement, the divergence between Flash Profile and Pivot Profile is more pronounced. This highlights the importance of selecting the appropriate method based on the research objective and the specific sensory characteristics of the product being analyzed.

## 4. Conclusions

This study compared three rapid descriptive methods (CATA, Flash Profile, and Pivot Profile) for evaluating consumer perception of muffins. The results demonstrated that each technique possesses specific strengths depending on the type of sensory attributes evaluated and the application context. The CATA method stood out for its simplicity, high discriminative power for textural and moisture-related attributes, and its strong agreement with Pivot Profile (RV = 0.741), making it an efficient tool for rapid industrial evaluation. Flash Profile, in contrast, enabled a more detailed characterization of complex attributes such as aroma and texture, although it showed lower consistency with the other methods. This suggests it captures distinct perceptions, possibly more influenced by the free vocabulary used by consumers. Finally, Pivot Profile excelled in capturing subtle and multidimensional sensory interactions, making it particularly useful when a holistic understanding of the product profile is desired. Taken together, the three methods provided complementary insights into the samples analyzed, all being suitable for consumer-based studies but offering different levels of descriptive depth. The choice of method is based on using Flash Profile when seeking a quick description due to a lack of information on sensory attributes; CATA when seeking to evaluate predefined attributes with many consumers in a simple and rapid manner; and Pivot Profile to compare new products or formulations against a reference sample. Therefore, the study underscores the importance of carefully selecting the appropriate sensory method, as the differences among them can significantly influence the interpretation and application of the results. Future research could include a broader range of flour blends and complementary techniques such as RATA or temporal analysis methods to further expand the sensory characterization of products made with alternative ingredients.

## Figures and Tables

**Table 1:** Data of participants in descriptive sensory tests.

Data	CATA *	Flash Profile	Pivot Profile
	*n* = 100	*n* = 80	*n* = 15
Gender (%)			
Male	47 (47.00)	43 (53.75)	07 (46.67)
Female	53 (53.00)	37 (46.25)	08 (53.33)
Age (%)			
18–30 years	84 (84.00)	53 (66.25)	12 (80.00)
31–39 years	14 (14.00)	21 (26.25)	02 (13.33)
40 years and older	02 (2.00)	06 (7.50)	01 (6.67)

* CATA: Check-All-That-Apply.

**Table 2:** Comparison of different descriptive methods using the RV coefficient.

Samples	CATA *		Flash Profile		Pivot Profile	
	F1	F2	F1	F2	F1	F2
T0	0.754	0.113	0.849	2.554	-	-
T1	0.161	−0.329	−2.167	1.517	0.246	0.064
T2	−0.251	−0.076	−0.557	−1.332	0.016	−0.042
T3	−0.198	0.077	−1.742	−0.965	−0.209	0.096
T4	−0.269	0.121	3.587	−0.351	−0.035	−0.002
T5	−0.244	0.056	0.031	−1.424	−0.034	−0.126
Coefficient RV						
CATA–Flash Profile	0.525					
Flash Profile–Pivot	0.249					
CATA–Pivot	0.741					

* CATA: Check all that apply. Note: T0 = control sample (100% wheat flour); T1 = 100% purple corn flour (PCF); T2 = 25% PCF/75% KF; T3 = 50% PCF/50% KF; T4 = 75% PCF/25% KF; and T5 = 100% kiwicha flour (KF).

## Data Availability

The original contributions presented in the study are included in the article; further inquiries can be directed to the corresponding authors.
